# Manipulation of Microglial Sialylation in Alzheimer’s Disease Mouse Models of Amyloid and Tau Pathology with Neuraminidase 1

**DOI:** 10.1002/alz.089265

**Published:** 2025-01-03

**Authors:** Caitlyn Fastenau, Sabrina Smith, Madison Bunce, Kevin F. Bieniek, Sarah C. Hopp

**Affiliations:** ^1^ UT Health San Antonio, San Antonio, TX USA; ^2^ Glenn Biggs Institute for Alzheimer’s & Neurodegenerative Diseases, University of Texas Health Sciences Center at San Antonio, San Antonio, TX USA; ^3^ Biggs Institute for Alzheimer’s & Neurodegenerative Diseases, UT Health San Antonio, San Antonio, TX USA

## Abstract

**Background:**

Glycosylation is the most common post‐translational modification in the brain. Aberrant glycosylation patterns are present in cerebrospinal fluid and brain tissue from Alzheimer’s disease (AD) patients. Specifically, dysregulation of a particular form of terminal glycoconjugate modification, sialylation, has been identified in AD. Terminal sialic acid modifications help facilitate important functions including cell‐to‐cell interaction, cell migration, and immune regulation. We have identified significantly increased α‐2,6 *N*‐linked sialylation of microglia proximal to amyloid β (Aβ) plaque pathology in post‐mortem human AD brains as well as of microglia during tauopathy. Yet we have limited understanding of the role of sialic acid residues on microglia in AD and other tauopathies.

**Methods:**

This study aims to determine if manipulation of α‐2,6 *N*‐sialylation in early stages of AD pathology can impact microglia interaction with the pathological proteins Aβ and tau. The present study investigates the biological consequence of administering neuraminidase 1, the cleavage enzyme for terminal α‐2,6 sialic acid from glycoconjugates in models of amyloid and tau pathology. We have validated Sambucus Nigra (SNA) plant‐derived lectin for labeling α‐2,6 *N*‐linked sialic acid residues in tissue and cells.

**Results:**

Our preliminary data demonstrate that incubation with exogenous neuraminidase 1 enzyme leads to the cleavage of terminal α‐2,6 sialic acid in BV2 murine microglia and on brain sections from post‐mortem human AD tissue. To test how neuraminidase 1 mediated α‐2,6 sialic acid cleavage affects microglial interactions with Aβ and tau, 4‐month‐old male and female 5XFAD, PS19, and wildtype mice received a single stereotaxic injection of neuraminidase 1 enzyme into the right cortex. 24 hours following injection, the mice were sacrificed, and their brains removed for histological analysis. We expect to visualize a significant decrease in α‐2,6 sialic acid levels in 5XFAD and PS19 mice following injection of neuraminidase 1 due to the increased likelihood of α‐2,6 cleavage. Additionally, we hypothesize that removal of terminal α‐2,6 sialic acids will increase microglia density near plaques and phosphorylated tau due to increased microglia phagocytic activity.

**Conclusion:**

These findings will advance the field of microglia glycobiology and provide further evidence for the functional role of sialylation for microglia response to both amyloid and tau pathology.

